# Habitual physical activity and sarcopenia: a systematic review and meta-analysis of prospective cohort studies

**DOI:** 10.7189/jogh.16.04095

**Published:** 2026-06-26

**Authors:** Dacheng Dai, Fangfang Xie, Jiahe Cui, Siyu Wang, Junhao Cai, Guimao Wang, Fei Yao

**Affiliations:** 1 Shanghai Municipal Hospital of Traditional Chinese Medicine, Shanghai University of Traditional Chinese Medicine, Shanghai, China; 2 School of Acupuncture-Moxibustion and Tuina, Shanghai University of Traditional Chinese Medicine, Shanghai, China

## Abstract

**Background:**

Habitual physical activity (HPA) has been associated with a lower risk of sarcopenia by enhancing skeletal muscle protein synthesis and suppressing systemic inflammation. However, the evidence for a long-term protective association remains inconclusive. Therefore, we conducted a systematic review and meta-analysis to quantify the association between HPA and sarcopenia.

**Methods:**

We searched PubMed, the Cochrane Library, EMBASE, Cumulative Index to Nursing and Allied Health Literature, Web of Science, and the China National Knowledge Infrastructure for prospective cohort studies on the relationship between physical activity (PA) and sarcopenia. We selected English and Chinese-language literature published before 6 October 2025, and assessed study quality using the Newcastle-Ottawa Scale. Data were statistically synthesised by calculating pooled relative risks (RRs) and 95% confidence intervals (CIs) using a random-effects model with the generic inverse-variance method.

**Results:**

This meta-analysis included nine prospective cohort studies involving 21 265 participants. High levels of HPA were associated with a significantly lower risk of sarcopenia compared to the low levels (RR = 0.55; 95% CI = 0.44–0.67). This protective association remained consistent in subgroup analyses stratified by gender and by compliance with international PA guidelines. Furthermore, moderate HPA was also associated with a reduced risk compared to low HPA levels (RR = 0.73; 95% CI = 0.50–0.96).

**Conclusions:**

Our analysis indicates that moderate to high levels of HPA are independently associated with a lower risk of sarcopenia, serving as a significant protective factor. However, given the methodological heterogeneity in PA measurement, further high-quality prospective studies are needed to clarify the optimal PA dose while accounting for potential reverse causality.

**Registration:**

PROSPERO: CRD420251162529.

Sarcopenia is an age-related progressive loss of muscle mass, decreased muscle strength, or impaired muscle function that affects 10–16% of older adults worldwide [1,2]. In terms of age groups, its prevalence is estimated at 5–13% among people aged 60–70 years and rises to 11–50% in those over 80 years [3]. The condition significantly increases the risk of falls, fractures, physical disability, and death, placing a huge economic burden on healthcare systems [4]. Lack of physical activity (PA) [5], malnutrition [6], smoking [7], excessive sleep duration [8], and diabetes [9] have been associated with an increased risk of sarcopenia. Gender has also been suggested as a key moderating variable, with a cross-sectional study showing that women have a higher risk of developing sarcopenia than men, and the protective effects of different intensities of PA may vary between genders [2].

Sarcopenia is managed and treated through interventions targeting nutrition or exercise, and in some cases, supplements or medication [10]. However, as there are no approved drugs for the condition, clinical intervention is still centred on physical rehabilitation, focusing on strengthening muscles and improving walking function through structured exercise training such as resistance training and gait retraining [11,12].

The World Health Organization (WHO) defines PA as ‛any physical activity that consumes energy and produces skeletal muscle’ [13]. Distinct from clinical exercise used for rehabilitation, habitual physical activity (HPA) is crucial for the primary prevention of sarcopenia [5,13]. As a lifestyle-oriented approach, HPA is easier for older adults to sustain. Physiologically, HPA counteracts muscle loss by stimulating protein synthesis, improving insulin sensitivity, and reducing chronic inflammation [1]. For adults to maintain optimal health, the WHO and the American College of Sports Medicine (ACSM) recommend a weekly volume of at least 150 minutes of moderate-intensity activity, or 600 metabolic equivalent of task (MET) minutes/week [13]. HPA encompasses an individual’s long-term behavioural patterns across four main domains: occupational (manual work), household (heavy chores), transportation (walking to destinations), and leisure time activities (gardening or brisk walking) [14]. Reduced PA significantly contributes to sarcopenia, creating a vicious cycle where a lack of activity leads to decreased strength, further reducing PA [15]. While systematic exercise training has been confirmed to improve function in affected patients, HPA may reduce the incidence of sarcopenia in healthy older adults by enhancing skeletal muscle protein synthesis and improving the anabolic hormonal environment [16].

Few meta-analyses have been conducted to assess the impact of PA on sarcopenia. One meta-analysis found that physically active people had an average 55% lower chance of developing sarcopenia later in life compared to those who were almost inactive [5]. However, the optimal dose and intensity of HPA required to prevent disease onset remain unclear. Therefore, we aimed to examine the association between various levels of HPA and the risk of incident sarcopenia to provide quantifiable data on the relationship between daily PA and preservation of muscle health.

## METHODS

We followed the PRISMA guidelines in reporting our findings and preregistered this systematic review on PROSPERO (CRD420251162529). Although we initially planned to include case-control studies, we ultimately restricted the synthesis to prospective cohort studies to establish a clearer temporal relationship and minimise recall bias for incident sarcopenia.

### Literature search

We systematically searched Embase, Cochrane Library, PubMed, Cumulative Index to Nursing and Allied Health Literature, Web of Science, and the China National Knowledge Infrastructure, from their respective inceptions to 6 October 2025. To ensure a comprehensive and reproducible search, we utilised a combination of controlled vocabulary (*e.g.* MeSH terms in PubMed and Emtree terms in Embase) and free-text keywords tailored to each platform (File S1 in the **Online Supplementary Document**). We manually reviewed the references of the included literature to supplement studies that met the criteria.

### Inclusion and exclusion criteria

Two researchers (DCD and FFX) independently screened the titles and abstracts of all retrieved records, followed by a full text review of potentially eligible articles. We included prospective cohort studies exploring the association between PA and sarcopenia, as long as they provided odds ratios (ORs), relative risks (RRs), or hazard ratios (HRs), reported RR estimates and their 95% confidence intervals (CIs), or provided the necessary data to calculate these indicators. We restricted inclusion to prospective cohort studies because our primary outcome was incident sarcopenia. Unlike retrospective designs, such as case-control studies, prospective cohorts allow for assessment of HPA prior to disease onset, thereby establishing a clear temporal relationship and minimising recall bias. In this study, we specifically define the exposure as HPA, which must be distinguished from structured clinical exercise interventions [14]. Specifically, HPA was defined by capturing activities across domains such as manual work, fast walking, or heavy housework using objective measures (*e.g.* accelerometers) or validated questionnaires (*e.g.* International Physical Activity Questionnaire (IPAQ), Japan Public Health Center – Physical Activity Questionnaire (JPHC-PAQ)).

We excluded studies focusing solely on structured clinical exercise interventions to minimise confounding between lifestyle exposure and medical prescriptions; studies that combined PA with other interventions such as diet, behavioural therapy, and antidepressants; and editorials, reviews, meta-analyses, commentaries, news articles, letters to the editor, or practice guidelines. If two researchers disagreed on the inclusion criteria, a third senior researcher (FY) participated in the discussion until a final consensus was reached.

### Data extraction

For the included studies [17–25], we systematically extracted the first author's last name, year of publication, study location, total sample size, number of sarcopenia cases, gender stratification (if applicable), PA domains, effect size (RR, OR, or HR) and 95% CIs under the maximally adjusted model, and the covariates adjusted by the model. To maximise evidence synthesis, for studies that did not directly report effect estimates, we attempted to manually derive risk estimates from raw contingency tables or incidence counts provided in the text. However, if essential statistical parameters such as 95% CIs or standard errors remained unavailable after these derivation attempts, we excluded those studies to maintain methodological rigour.

To address clinical heterogeneity in PA metrics (*e.g.* kcal/week, daily steps, or frequency), we estimated standardised MET-hours/week equivalents where sufficient duration and intensity data were available. We further categorised studies as standard-compliant or below standard based on whether their highest PA group met the WHO/ACSM benchmark (≥150-minute/week or 600 MET-min/week). We extracted the effect sizes as defined by the authors of the included studies for the high and low HPA groups and used them in our primary meta-analysis. To investigate dose-response relationships, we extracted data comparing the moderate and low HPA groups (if reported) for secondary analysis.

### Study quality

Two researchers (JHC and SYW) independently assessed the quality of the studies using the Newcastle-Ottawa Study Scale (NOS). In case of discrepancies in scores, a third senior researcher (FY) made the final decision. The NOS maximum score is 9 points and covers three main areas: selection, comparability, and outcome (cohort studies). A score of 0–4 indicates low quality, and 5–9 indicates high quality [26].

### Data analysis

We quantified the association between HPA and sarcopenia incidence using pooled effect estimates. For this purpose, we extracted the effect size (RR, HR, or OR) and its 95% CI between the high and the low HPA group from the maximally adjusted model of each study for the primary analysis. In accordance with established meta-analysis practices for prospective cohorts, HRs were treated as equivalent to RRs. To address the potential bias from the non-interchangeability of different effect measures (particularly ORs when the outcome is not rare), we conducted a stratified sensitivity analysis by grouping studies according to their original measure type (OR *vs.* RR/HR) to evaluate the stability of our findings. We manually derived risk estimates for studies that provided sufficient raw data, but no adjusted estimates. All effect sizes and their CIs were log-transformed to obtain log effect sizes. To ensure statistical precision, standard errors (SEs) were derived from the log-transformed 95% CIs using the following formula: SE = (ln(upper limit) − ln(lower limit))/3.92.

We used a random effects model to combine the weighted average of the log effect sizes using the generic inverse-variance method. To explore sources of heterogeneity, we pre-specified subgroup analyses based on gender (men/women/mixed) and effect measure type (OR *vs.* RR/HR). To assess publication bias and small-study effects, we plotted funnel plots and combined Begg's rank correlation test and Egger's linear regression test. If asymmetry was found, we used the trim-and-fill method to assess its impact on the pooled effect size.

Furthermore, we conducted subgroup analysis by sex, excluded the study by Shephard *et al*. [24] for a sensitivity analysis, and performed a secondary moderate-versus-low analysis as exploratory *post hoc* analyses to further elucidate heterogeneity and the dose-response relationship identified during data extraction.

All statistical analyses were performed using Stata 18.0 software (Stata Corp LP, College Station, Texas, USA). All statistical tests were two-tailed, and *P* < 0.05 was considered statistically significant.

## RESULTS

### Study selection process

We initially retrieved 28 691 records from all databases and did not identify additional ones through reference list screening. After removing duplicates, we retained 11 220 independent records for title/abstract screening. We excluded 11 138 records that did not meet our criteria, leaving 82 for full-text eligibility assessment. We further excluded 73 records, as 57 had insufficient data for meta-analysis (including 35 lacking incident sarcopenia risk estimates, 14 using non-standardised sarcopenia definitions, and 8 missing 95% CIs or SEs), 24 were not prospective cohort studies, and five were not written in English or Chinese. Finally, we included nine studies that met all inclusion criteria for a qualitative and quantitative synthesis (Figure S1 in the **Online Supplementary Document**).

### Characteristics of included studies

This meta-analysis ultimately included nine longitudinal cohort studies, four of which provided stratified effect sizes by sex [19,22–24], providing a total of 13 independent data points for our analysis (Table S1 in the **Online Supplementary Document****)**. These nine studies comprised a total of 21 265 participants distributed across seven countries. Studies from Asia accounted for the majority of this sample (three from Japan [19,24,25], one from South Korea [23], and one from China [21], with the remaining studies conducted in the USA [18], the UK [22], Iceland [20], and Sweden [17]). All studies adjusted for key confounding factors in their statistical models. Age and sex (in non-stratified studies) were the most basic adjustment variables. Most studies also adjusted for body mass index (BMI), smoking status, alcohol consumption, education level, and history of chronic diseases (such as diabetes and hypertension).

We found significant clinical heterogeneity regarding the definitions of exposure (PA) and outcome (sarcopenia) across studies. First, regarding the assessment of PA, there was a high degree of consistency in capturing habitual patterns across multiple domains. Six of the included studies [18–21,23,24], utilised instruments (*e.g.* IPAQ, JPHC-PAQ, or validated checklists) that specifically recorded non-leisure activities such as manual work, commuting, and heavy chores. Second, the criteria for PA level classification varied, including kcal/week, MET-hour/d, min/week, or weekly frequency. Finally, regarding the definition of sarcopenia, Yang *et al.* [22] used only low grip strength as a diagnostic criterion (*i.e.* possible sarcopenia), Shephard *et al.* [24] used a unique criterion relative to the healthy young population, and the remaining studies [17–21,23,25] adopted the low muscle mass and low muscle strength/function criterion based on the Asian Working Group for Sarcopenia/European Working Group on Sarcopenia in Older People (AWGS/EWGSOP) consensus.

### Primary outcome: HPA (high *vs.* low) and the risk of sarcopenia

Of the nine included studies, seven [17–23] directly reported the effect size (OR, HR, or RR) and 95% CIs of the highest HPA group compared with the lowest HPA group, and we extracted these values as reported. Two studies [24,25] used the highest HPA group as the reference; we recalculated them to use the lowest HPA group as the reference by taking the reciprocal (RR = 1/original RR). For studies with more than three HPA exposure levels, we used the highest quartile (Q4) as the high HPA group and the lowest quartile (Q1) as the low HPA group [19,24].

We used a random-effects model to perform a meta-analysis of 13 effect sizes extracted from nine studies. Forest plot results (Figure S2 in the **Online Supplementary Document**) showed a significant inverse association between HPA and the risk of incident sarcopenia. Compared with the low HPA group, the high HPA group was associated with a 45% lower risk of incident sarcopenia (RR = 0.55, 95% CI = 0.44–0.67).

Of the 13 effect sizes, nine showed statistically significant protective associations. The remaining four effect sizes had CIs that crossed the null threshold of 1.0, with the point estimate (RR = 1.38) for one effect size showing the opposite trend. We used the *I*^2^ statistic to assess heterogeneity among studies, with results indicating moderate to high heterogeneity (*I*^2^ = 60.8%, *P* = 0.002). Thus, we conducted subgroup analyses by gender to investigate the source of this overall heterogeneity (Figure S3 in the **Online Supplementary Document**). We noted that HPA had a significant protective association among women (RR = 0.44; 95% CI = 0.28–0.59), the men subgroup (RR = 0.59; 95% CI = 0.29 − 0.90) and the mixed-sex subgroup (RR = 0.65; 95% CI = 0.55–0.76). There was no heterogeneity within the mixed-sex subgroup (*I*^2^ = 0.0%, *P* = 0.427), while males showed higher heterogeneity (*I*^2^ = 66.2%, *P* = 0.031). The intergroup heterogeneity test result (*P* = 0.067) indicated that gender grouping failed to explain the significance of overall heterogeneity among studies.

We conducted a leave-one-out sensitivity analysis to test the robustness of the results and found that the pooled effect size did not change significantly after each study was successively removed (Figure S4 in the **Online Supplementary Document**). All newly calculated pooled RR values remained between 0.477 (after excluding the study by Murphy *et al.* [18]) and 0.560 (excluding the study by Shephard *et al.* [24]), and their 95% CIs remained statistically significant (all less than 1.0).

Despite the robustness of our findings, the overall heterogeneity remained high. We conducted an additional sensitivity analysis by excluding the study by Shephard *et al*. [24], which exhibited significant methodological divergence by using objective accelerometers for continuous 24-hour monitoring over five years (analysing representative annual windows). The overall heterogeneity (*I*^2^) significantly decreased from 60.8% to 15.0% (*P* = 0.301), with a pooled RR of 0.61 (95% CI = 0.53–0.70) (Figure S5 in the **Online Supplementary Document**), suggesting it to be the primary source of statistical heterogeneity.

To further address concerns regarding the harmonisation of HPA exposures, we performed an additional subgroup analysis based on compliance with international PA guidelines (WHO/ACSM). This subgroup analysis showed that the protective association was nearly identical between studies strictly compliant with international guidelines (RR = 0.56; 95% CI = 0.40–0.72) and those using lower thresholds (RR = 0.55, 95% CI = 0.43–0.67; *P* = 0.921) (Figure S6 in the **Online Supplementary Document**).

To address the non-interchangeability of effect measures, we stratified the analysis by measure type (Figure S7 in the **Online Supplementary Document**). Here, HPA maintained a significant protective association in both the OR subgroup (pooled RR = 0.62, 95% CI = 0.53–0.72) and the RR/HR subgroup (pooled RR = 0.38; 95% CI = 0.24–0.52). Although the subgroup differences in effect magnitude were statistically significant (*P* = 0.006), the consistent direction and significance across both groups reinforce the fundamental robustness of the results.

Further subgroup analysis focused on addressing definition variability confirmed significant protective associations across all diagnostic frameworks, with heterogeneity nearly eliminated within the consensus-based (AWGS/EWGSOP) group (*I*^2^ = 1.8%, *P* = 0.419) (Figure S8 in the **Online Supplementary Document**). The results remained robust despite significant inter-group differences (*P* < 0.01) driven by varying definition thresholds.

Regarding publication bias, the funnel plot showed slight asymmetry and Begg's test found no significant bias *(P =* .067), but Egger's test indicated statistically significant funnel plot asymmetry (*P* = 0.038) (Figures S9–11 in the **Online Supplementary Document**). This suggests potential publication bias or small-study effects, meaning the pooled effect size (RR = 0.55) might slightly overestimate the true magnitude of the association.

Due to the Egger’s test result, we performed a trim-and-fill analysis, which estimated and filled in four hypothetical missing studies (Figure S12 in the **Online Supplementary Document**). The adjusted pooled RR (17 studies) was 0.485 (95% CI = 0.375–0.594), which remained significantly less than 1.0, confirming that the inverse association between HPA and sarcopenia risk is reliable even after accounting for potential bias.

### Secondary outcome: HPA (moderate *vs.* low) and the risk of sarcopenia

To investigate the dose-response relationship, we assessed the protective association of moderate-level HPA compared to low-level HPA [18,20,22]. Moderate HPA was associated with a 27% lower risk of incident sarcopenia compared to low HPA (pooled RR = 0.73; 95% CI = 0.50–0.96; *I*^2^ = 72.7%) (Figure S13 in the **Online Supplementary Document**). However, these findings should be considered exploratory rather than confirmatory, given the small number of contributing studies and the high heterogeneity observed. Furthermore, the definitions of moderate HPA varied significantly across the included studies, which precludes a definitive conclusion regarding a graded dose-response relationship at this stage.

### Research quality evaluation

All nine cohort studies received NOS scores ranging from 7 to 9 stars, with a mean score of 8.2 stars (median (MD) = 8, standard deviation (SD) = 0.67), indicating high overall quality (Table S2 in the **Online Supplementary Document**). All studies received a perfect score (2 stars) in the comparability dimension, demonstrating that they adequately adjusted for key confounding factors, such as age, BMI, and smoking status. The main deductions were due to high follow-up loss rates in some studies (*e.g.* Nishimoto *et al.* [25] (36% loss to follow-up) and Yang *et al.* [22] (34% loss to follow-up)) or failure to assess baseline outcomes (Wakana *et al.* [19]).

## DISCUSSION

As a cost-effective strategy for combatting age-related muscle weakness [27], PA is crucial for preventing physical decline and disability caused by muscle atrophy and for ensuring the elderly's ability to live independently [28]. The EWGSOP points out that patients with sarcopenia experience significant declines in grip strength, knee extension, and knee flexion strength [15]. Therefore, improving muscle strength is crucial for patients with sarcopenia and can effectively prevent further development of the disease. According to ACSM and the American Heart Association recommendations on exercise and PA for the elderly, the commonly used and effective exercise prescriptions for elderly patients with sarcopenia mainly include two categories of PA: aerobic and resistance [29,30]. For adults to maintain optimal health, the recommended weekly exercise volume is no less than 150 minutes of moderate-intensity activity or the equivalent of 75 minutes of vigorous-intensity activity [13].

It is crucial to distinguish HPA from structured exercise training. For instance, Choe *et al.* [23] demonstrated that, while structured resistance training (≥2 times/week) was a major modifiable factor for preventing sarcopenia in men, the association with general PA levels was more modest. This suggests that HPA may protect against sarcopenia through different pathways, such as maintaining a higher reserve of muscle mass and strength throughout the ageing process [18,22]. Given the observational nature of the included prospective cohorts, the inverse associations identified in our study should be interpreted as protective correlations, rather than definitive causal effects. A critical caveat is the potential for reverse causality that individuals in subclinical stages of sarcopenia or with baseline frailty may naturally reduce their physical activity, a sick quitter effect that could inflate the apparent protective association [15].

Our core finding concerns the protective association of habitual physical activity against sarcopenia. Specifically, our primary analysis (high *vs.* low HPA) robustly confirmed a 45% risk reduction (RR = 0.55; 95% CI = 0.44–0.67). This conclusion demonstrated high robustness across our subgroup analyses, leave-one-out sensitivity analysis, and trim-and-fill bias correction (adjusted RR = 0.485). We also conducted a secondary analysis comparing moderate *vs.* low HPA (RR = 0.73; 95% CI = 0.50–0.96); yet as it included only four studies and was accompanied by extremely high and significant statistical heterogeneity (*I*^2^ = 72.7%, *P* = 0.012), the robustness of its conclusions is limited. Thus, the strongest public health implication from this meta-analysis is that maintaining high levels of HPA represents a protective strategy against sarcopenia development compared to a sedentary lifestyle (low HPA), consistent with health guidelines from authoritative bodies.

We initially hypothesised that the observed high statistical heterogeneity in our sample might stem from gender differences. However, subgroup analysis results refuted this assumption, as it revealed that the protective effect of HPA was consistently and significantly present among women (RR = 0.44), men (RR = 0.59), and mixed-sex subgroup (RR = 0.65). Although the point estimates for women were slightly stronger than those for men, the test for between-group heterogeneity did not reach statistical significance, indicating that gender was not the primary driver of inconsistent results. This finding aligns with current international consensus recommendations [13,15,27] that physical inactivity represents a core risk factor common to both genders, and that increasing HPA serves as a key protective factor within this population. Thus, despite numerical variations in effect size estimates across certain studies, the conclusion that HPA may have protective association against sarcopenia for both men and women is supported by the existing evidence base.

Further sensitivity analysis identified methodological diversity as the primary driver of the observed high heterogeneity. Specifically, when excluding Shephard *et al.* [24] – the most methodologically divergent study due to its use of objective accelerometers for continuous 24-hour monitoring over five years (analysing representative annual windows) – overall heterogeneity dropped from an *I*^2^ of 60.8% to 15.0%, while the pooled effect remained robust (RR = 0.61). This confirms that the observed variation stems not from poor study quality or conflicting results, but from high-quality studies employing different measurement rules. To address the challenge of varying categorical definitions, we standardised PA levels into estimated MET-hours/week, which confirmed a clear biological gradient within each cohort. While absolute thresholds varied across studies, the consistent protection observed in our guideline-based subgroup analysis suggests that a relative increase in HPA within a specific population serves as a protective factor for sarcopenia.

Additionally, stratified sensitivity analysis identified the use of diverse statistical indicators as a significant source of heterogeneity (*P* = 0.006). While the RR/HR subgroup showed a stronger protective effect (0.38) than the OR subgroup (0.62), both consistently demonstrated significant protective associations. This directional consistency reinforces the robustness of the link between physical activity and reduced sarcopenia risk. To address the variability in sarcopenia definitions, we conducted a subgroup analysis stratified by diagnostic framework. The near-elimination of heterogeneity (*I*^2^ = 1.8%) within the consensus-based subgroup (AWGS/EWGSOP) provides compelling evidence that HPA is consistently associated with a lower risk of incident sarcopenia when standardised criteria are applied.

Our findings have substantial biological plausibility. PA can induce muscle protein synthesis by activating satellite cells to increase myofibrillar protein synthesis [31] and reduce intramuscular fat infiltration [32], thereby conferring benefits to muscle mass, strength, and function [33]. Aerobic exercise, involving rhythmic contraction of large muscle groups, serves as an effective stimulus for inducing both central adaptations (*e.g.* mitochondrial biogenesis) and peripheral adaptations (*e.g.* capillary neogenesis) in skeletal muscle [34]. Its core physiological mechanism lies in significantly increasing mitochondrial density and function by activating key signalling pathways such as peroxisome proliferator-activated receptor gamma coactivator 1-Alpha [35,36], thereby enhancing energy production capacity. Simultaneously, exercise promotes increased capillary density within skeletal muscle through factors like vascular endothelial growth factor [37], greatly optimising oxygen delivery and uptake efficiency in muscle tissue. These adaptive changes collectively manifest as a significant improvement in muscular endurance and overall aerobic capacity, meeting the heightened demands for energy and oxygen flux during exercise [38,39].

The strength of this study lies in the inclusion of high-quality prospective cohort studies. However, limitations remain, including potential language bias and residual confounding. The observed associations may reflect broader healthy lifestyle patterns, including diet quality, socioeconomic status, and comorbidity burden, where PA serves as a marker of overall health rather than a singular causal factor. The secondary analysis comparing moderate *vs*. low HPA should be treated as exploratory, given the high heterogeneity and the small number of studies. These factors render the dose–response conclusion for moderate *vs.* low HPA unstable and preclude us from proposing a precise optimal dose of PA. Future research should prioritise prospective cohorts with repeated PA assessments and better baseline phenotyping, such as objective accelerometers, to more rigorously isolate the causal role of PA in lowering sarcopenia risk.

## CONCLUSIONS

This systematic review and meta-analysis indicates that moderate to high levels of HPA are independently associated with a lower risk of sarcopenia. However, the magnitude of this protective association varies across studies, largely due to substantial methodological heterogeneity in HPA measurement and sarcopenia diagnostic criteria, which is a factor contributing to the high statistical heterogeneity observed. Therefore, future high-quality prospective studies are urgently needed to further clarify the dose-response relationship (optimal frequency, intensity, and duration) of HPA while accounting for potential reverse causality. These studies should adopt standardised measurement tools and diagnostic criteria to yield more robust and definitive conclusions.

## Additional material


Online Supplementary Document
