# Supplementary material for: Habitual physical activity and sarcopenia: a systematic review and meta-analysis of prospective cohort studies
Source: J Glob Health. 2026 Jun 26;16:04095. doi: 10.7189/jogh.16.04095 (PMC13307538; doi:10.7189/jogh.16.04095)
Supplement: Online Supplementary Document [file jogh-16-04095-s001.pdf]

**Supplement to: Dai D, Xie F, Cui J, Wang S, Cai J, Wang G, Yao F. Habitual physical activity and sarcopenia: a systematic review and meta-analysis of prospective cohort studies. J Glob Health. 2026;16:04095.**

1. Full search strategy
2. Characteristics of the 9 studies on habitual physical activity and sarcopenia risk (Table S1)
3. Quality of studies according to Newcastle Ottawa Scale (Table S2)
4. Figures

## File S1. Full search strategy

Last Search Date: October 6, 2025

Total Databases: 6 (PubMed, Embase, Cochrane Library, Web of Science, CINAHL, CNKI)

Filters Applied: Humans; English language (except for CNKI which used Chinese).

1. PubMed Search Strategy:

Search Terms: ( ( ("Exercise"[Mesh]) OR ( ( Exercises[Title/Abstract] OR "Exercise, Physical"[Title/Abstract] OR "Exercises, Physical"[Title/Abstract] OR "Physical Exercise"[Title/Abstract] OR "Physical Exercises"[Title/Abstract] OR "Exercise, Aerobic"[Title/Abstract] OR "Aerobic Exercise"[Title/Abstract] OR "Aerobic Exercises"[Title/Abstract] OR "Exercises, Aerobic"[Title/Abstract] OR "Exercise, Isometric"[Title/Abstract] OR "Exercises, Isometric"[Title/Abstract] OR "Isometric Exercises"[Title/Abstract] OR "Isometric Exercise"[Title/Abstract] OR "Acute Exercise"[Title/Abstract] OR "Acute Exercises"[Title/Abstract] OR "Exercise, Acute"[Title/Abstract] OR "Exercises, Acute"[Title/Abstract] OR "Exercise Training"[Title/Abstract] OR "Exercise Trainings"[Title/Abstract] OR "Training, Exercise"[Title/Abstract] OR "Physical Activity"[Title/Abstract] OR "Activities, Physical"[Title/Abstract] OR "Activity, Physical"[Title/Abstract] OR "Physical Activities"[Title/Abstract] ) ) ) AND ( ("Sarcopenia"[Mesh]) OR "Sarcopenias"[Title/Abstract] ) )

Result: 2,172

2. Cochrane Library Search Strategy:

Search Terms: ([mh Exercise] OR (Exercise OR "Physical activity" OR Exercises OR "Activities Physical" OR "Activity Physical" OR "Physical Activities" OR "Exercise Physical" OR "Exercises Physical" OR "Physical Exercise" OR "Physical Exercises" OR "Acute Exercise" OR "Acute Exercises" OR "Exercise Acute" OR "Exercises Acute" OR "Exercise Isometric" OR "Exercises Isometric" OR "Isometric Exercises" OR "Isometric Exercise" OR "Exercise Aerobic" OR "Aerobic Exercise" OR "Aerobic Exercises" OR "Exercises Aerobic" OR "Exercise Training" OR "Exercise Trainings" OR "Training Exercise" OR "Trainings Exercise"):ti,ab,kw) AND ([mh Sarcopenia] OR (Sarcopenia OR Sarcopenias):ti,ab,kw)

Result: 1,895

3. Web of Science Search Strategy:

Search Terms: (TS=("Exercise" OR "Exercise, Physical" OR "Exercises, Physical"OR "Physical Exercise"OR "Physical Exercises"OR "Exercise, Aerobic" OR "Aerobic Exercise" OR "Aerobic Exercises" OR "Exercises, Aerobic" OR "Exercise, Isometric"OR "Exercises, Isometric" OR "Isometric Exercises" OR "Isometric Exercise" OR "Acute Exercise" OR "Acute Exercises" OR "Exercise, Acute" OR "Exercises, Acute" OR "Exercise Training"OR "Exercise Trainings" OR "Training, Exercise" OR

"Physical Activity" OR "Activities, Physical" OR "Activity, Physical"OR "Physical Activities")) AND TS=("Sarcopenia" OR "Sarcopenias" )

Result: 7,128

4. Embase Search Strategy:

Search Terms: ('exercise'/exp OR 'exercise':ti,ab OR 'physical exercise':ti,ab OR 'aerobic exercise':ti,ab OR 'isometric exercise':ti,ab OR 'acute exercise':ti,ab OR 'exercise training':ti,ab OR 'physical activity':ti,ab) AND ( 'sarcopenia'/exp OR 'sarcopenia':ti,ab)

Result: 8,737

5. CINAHL Search Strategy:

Search Terms: ((MH "Exercise") OR (TI exercise OR AB exercise OR TI "physical exercise" OR AB "physical exercise" OR TI "aerobic exercise" OR AB "aerobic exercise" OR TI "isometric exercise" OR AB "isometric exercise" OR TI "acute exercise" OR AB "acute exercise" OR TI "exercise training" OR AB "exercise training" OR TI "physical activity" OR AB "physical activity")) AND ((MH "Sarcopenia") OR (TI sarcopenia OR AB sarcopenia))

Result: 7,500

6. China National Knowledge Infrastructure (CNKI)

Search Terms: (肌少症 OR 肌肉减少症) AND (体力活动 OR 运动 OR 锻炼 OR 身体活动)

Results: 1,259

Table S1. Characteristics of the 9 studies on habitual physical activity and sarcopenia risk included in the meta-analysis

| Authors, year | Gender | Region | Relative Risk (95%CI) for moderate vs low PA | Low PA defined by | Moderate PA defined by | High PA defined by | Adjustment factors (excluding age, sex) | PA Domains | Estimated MET-h/wk (low PA) | Estimated MET-h/wk (moderate PA) | Estimated MET-h/wk (high PA) | WHO/ACSM Alignment |
|---|---|---|---|---|---|---|---|---|---|---|---|---|
| Murphy RA, et al. 2014 | Men and women | USA | 0.87 (0.70, 1.06) | <500 kcal/wk | 500-1,499 kcal/wk | >1,500 kcal/wk | Race, smoking, BMI, self-reported health, pain, knee pain, diabetes, insulin, free testosterone, IL-6, TNF-α | Leisure, Transportation (Walking) | <7.1 | 7.1–21.4 | >21.4 | Exceeds Standard |
| Nishimoto K, et al. 2024 | Men and women | Japan | Not Reported | Applicable to one or more of five low-activity items (e.g., not walking, not exercising, not going out) | Not Reported | Reference group (i.e., not in the low-PA group; passed all 5 PA items) | Education level, medication, current smoking, alcohol consumption, cognitive activity, social activity | Leisure, Transportation, Functional | Inconvertible | NR | Inconvertible | Functional Status |
| Yang L, et al. 2019 | Men | UK | 0.44 (0.27, 0.67) | 1–3 times a month and hardly ever/never | once a week | >once a week | Smoking, alcohol, wealth, depressive symptoms, activity limiting illness, central obesity, diabetes | Leisure, General Lifestyle | Inconvertible | Inconvertible | Inconvertible | Below Standard |
| Yang L, et al. 2019 | Women | UK | 0.85 (0.55, 1.22) | 1–3 times a month and hardly | once a week | >once a week | Smoking, alcohol, wealth, depressive symptoms, activity limiting | Leisure, General Lifestyle | Inconvertible | Inconvertible | Inconvertible | Below Standard |

| | | | | | | | | | | | | |
|---|---|---|---|---|---|---|---|---|---|---|---|---|
| | | | | ever/never | | | illness, central obesity, diabetes | | | | | |
| Mijnarends DM, et al. 2016 | Men and women | Iceland | 0.79 (0.54, 1.14) | Non-exercise | <1 h MVPA/week | ≥1 h MVPA/week | Education, marital status, BMI, smoking status, total number of comorbidities, depressive symptoms, weight loss, cognitive function | Leisure, Household (Heavy housework) | ≈0 | <4.0 | ≥4.0 | Below Standard |
| Wakana S, et al. 2025 | Men | Japan | Not Reported | Total PA: <35.2 MET-hr/d | Not Reported | Total PA: ≥52.4 MET-hr/d | Marital status, education, occupation, BMI, energy intake, smoking, drinking, history of stroke, history of diabetes | Occupational, Household, Transportation, Leisure | <246.4 | NR | ≥366.8 | Far Exceeds |
| Wakana S, et al. 2025 | Women | Japan | Not Reported | Total PA: <35.0 MET-hr/d | Not Reported | Total PA: ≥46.4 MET-hr/d | Marital status, education, occupation, BMI, energy intake, smoking, drinking, history of stroke, history of diabetes | Occupational, Household, Transportation, Leisure | <245.0 | NR | ≥324.8 | Far Exceeds |
| Choe HJ, et al. 2022 | Men | South Korea | Not Reported | Low physical activity (<600 MET-min/week) | Not Reported | Moderate to high physical activity (≥600 MET-min/week) | Charlson comorbidity index, MNA-SF, resistance training, Ln(HOMA-IR), HbA1c | General HPA (IPAQ), Structured RT | <10.0 | NR | ≥10.0 | Fully Aligned |
| Choe HJ, et al. 2022 | Women | South Korea | Not Reported | Low physical activity (<600 MET- | Not Reported | Moderate to high physical activity (≥600 | Charlson comorbidity index, MNA-SF, resistance training, | General HPA (IPAQ), Structured RT | <10.0 | NR | ≥10.0 | Fully Aligned |

| | | | | | | | | | | | | |
|---|---|---|---|---|---|---|---|---|---|---|---|---|
| | | | | min/week) | | MET-min/week) | Ln(HOMA-IR), HbA1c | | | | | |
| Chang L, et al. 2025 | Men and women | China | Not Reported | Inactive (Nonleisure-time PA) | Not Reported | Active (Nonleisure-time PA, ≥600 MET-min/week) | BMI, ALB, protopathy, nPAN, P, BUN, WC, HGS, SMM | Occupational, Household, Transportation, Leisure | <10.0 | NR | ≥10.0 | Fully Aligned |
| Trevisan C, et al. 2022 | Men and women | Sweden | Not Reported | Inactive (≤2-3 times/month) | Not Reported | Active (≥1 time/week) | Education, smoking habits, alcohol consumption, MMSE, BMI, number of chronic diseases | Leisure, General Lifestyle | Inconvertible | NR | Inconvertible | Below Standard |
| Shephard RJ, et al. 2013 | Men | Japan | Not Reported | <7.8 min/d (>3 METs) (Q1) | Not Reported | >27.9 min/d (>3 METs) (Q4) | Baseline lean tissue mass, smoking status, alcohol intake | Comprehensive HPA (All movement) | <3.6 | NR | >13.0 | Exceeds Standard |
| Shephard RJ, et al. 2013 | Women | Japan | Not Reported | <7.9 min/d (>3 METs) (Q1) | Not Reported | >22.0 min/d (>3 METs) (Q4) | Baseline lean tissue mass, smoking status, alcohol intake | Comprehensive HPA (All movement) | <3.7 | NR | >10.3 | Exceeds Standard |

**Table S2 Quality of studies according to Newcastle-Ottawa Scale**

| First author, Year, Region | Selection (Max, score 4) | Comparability (Max, score 2) | Outcome (cohort) (Max, score 3) | Total Score (Max, 9) |
|---|---|---|---|---|
| Murphy RA, et al., 2014, USA | 4 | 2 | 3 | 9 |
| Nishimoto K, et al., 2024, Japan | 4 | 2 | 2 | 8 |
| Yang L, et al., 2019, UK | 4 | 2 | 2 | 8 |
| Mijnarends DM, et al., 2016, Iceland | 4 | 2 | 2 | 8 |
| Wakana S, et al., 2025, Japan | 3 | 2 | 2 | 7 |
| Choe HJ, et al., 2022, South Korea | 4 | 2 | 3 | 9 |
| Chang L, et al., 2025, China | 3 | 2 | 3 | 8 |
| Trevisan C, et al., 2022, Sweden | 4 | 2 | 3 | 9 |
| Shephard RJ, et al., 2013, Japan | 3 | 2 | 3 | 8 |

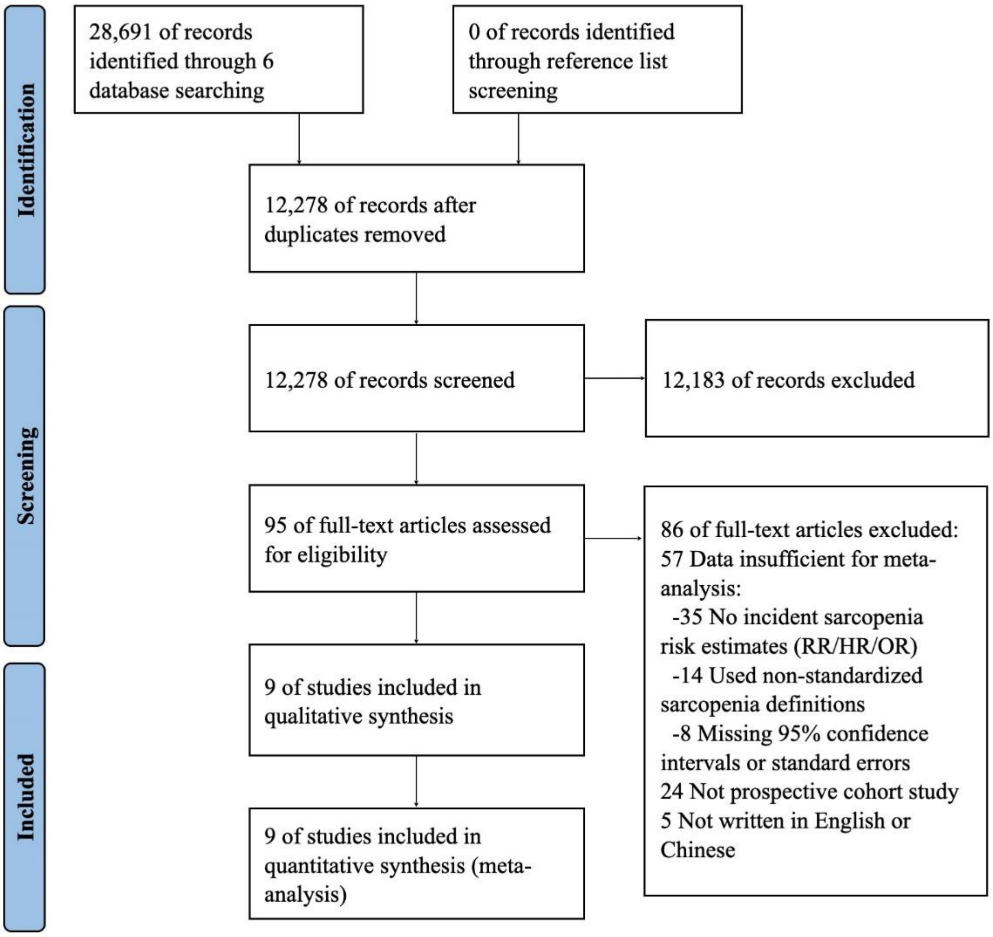


**Figure S1 PRISMA flow diagram**

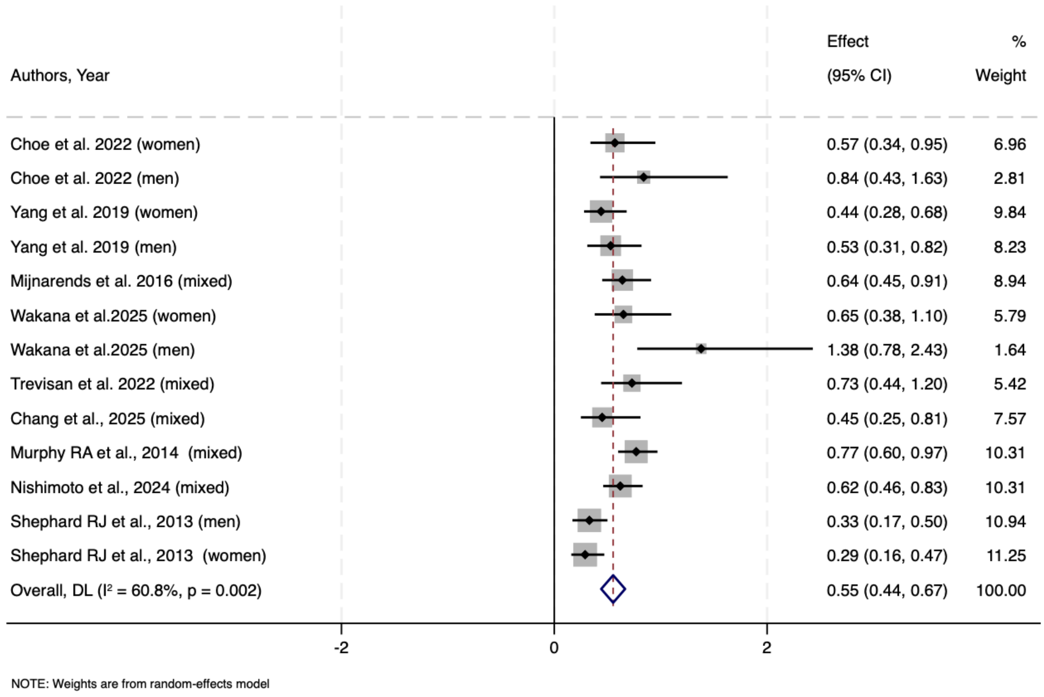


**Figure S2: Forest plot of a random effects meta-analysis including 13 risk estimates of sarcopenia for a high versus low level of HPA**

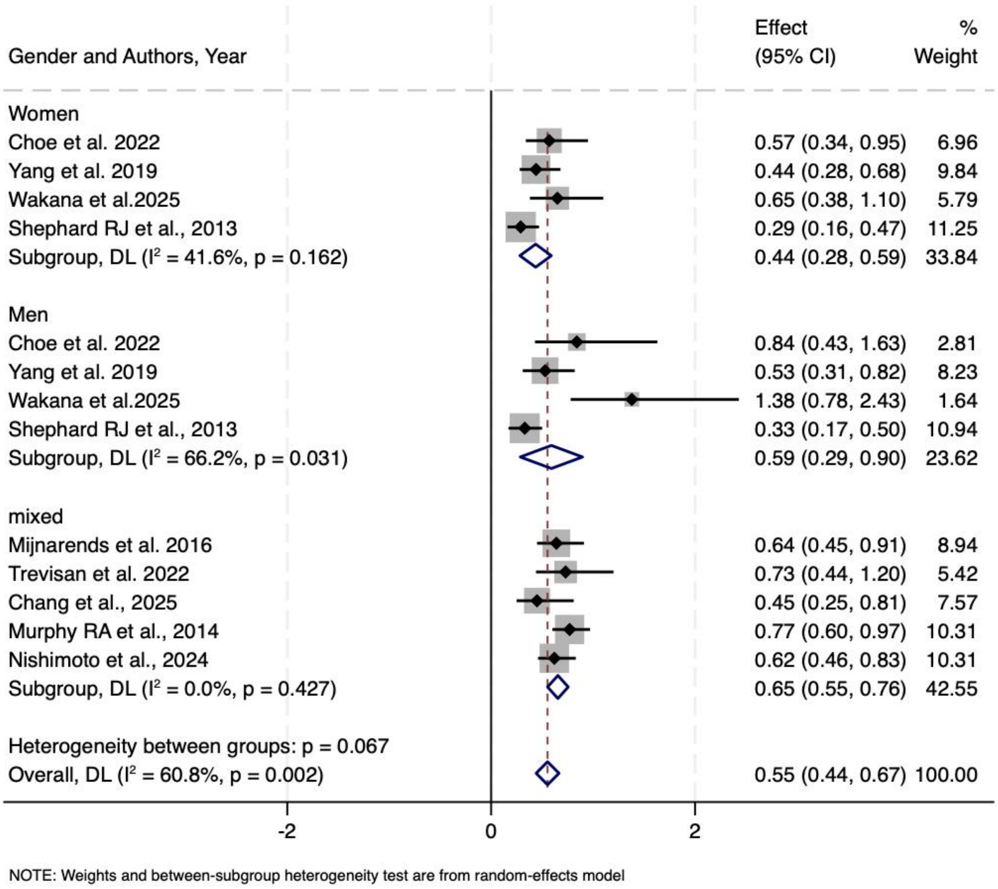


**Figure S3: Forest plot of a random effects meta-analysis including 13 risk estimates of sarcopenia for a high versus low level of HPA, grouped by gender**

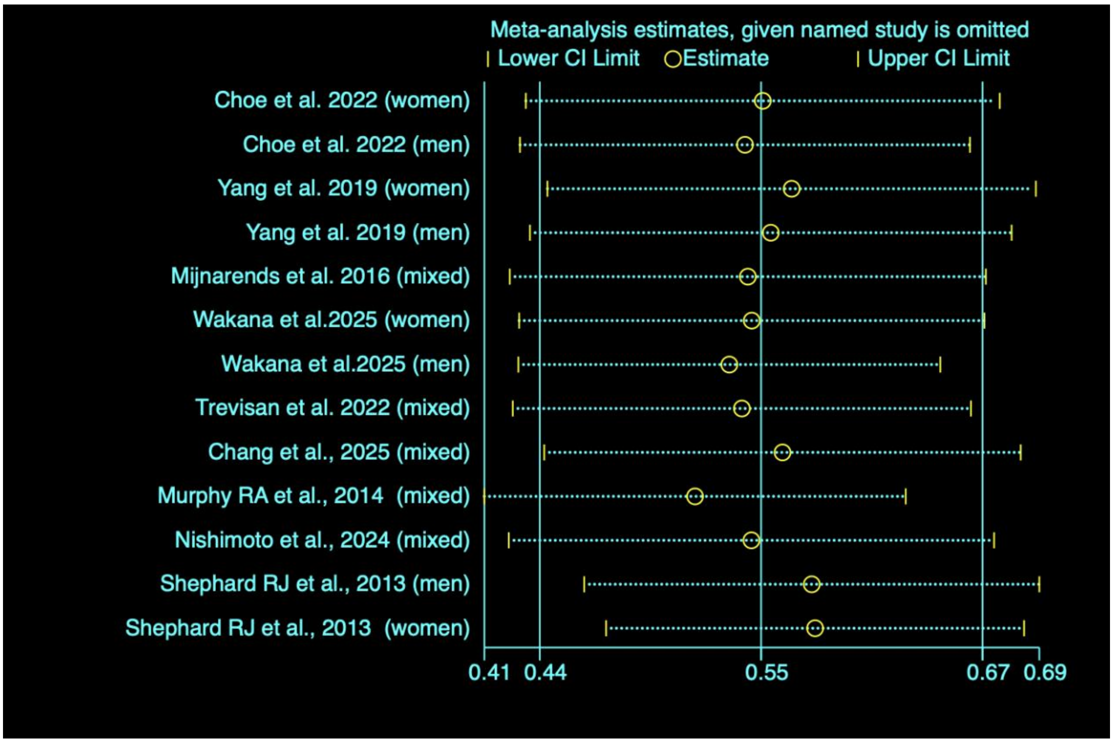


**Figure S4: Leave-one-out sensitivity analysis corresponding to the main random-effects meta-analysis**

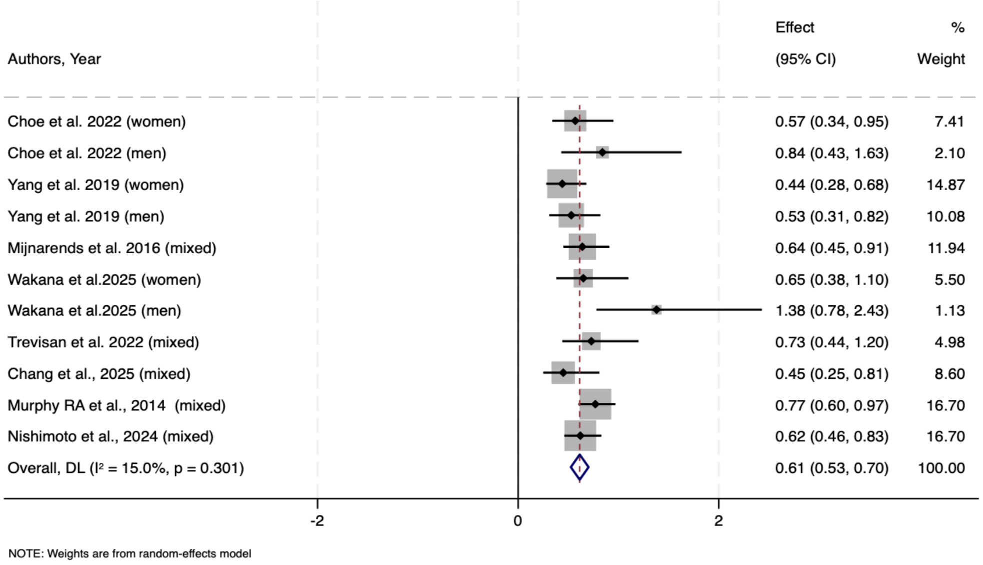


**Figure S5: Sensitivity analysis corresponding to the main random-effects meta-analysis, after excluding the studies by Shephard**

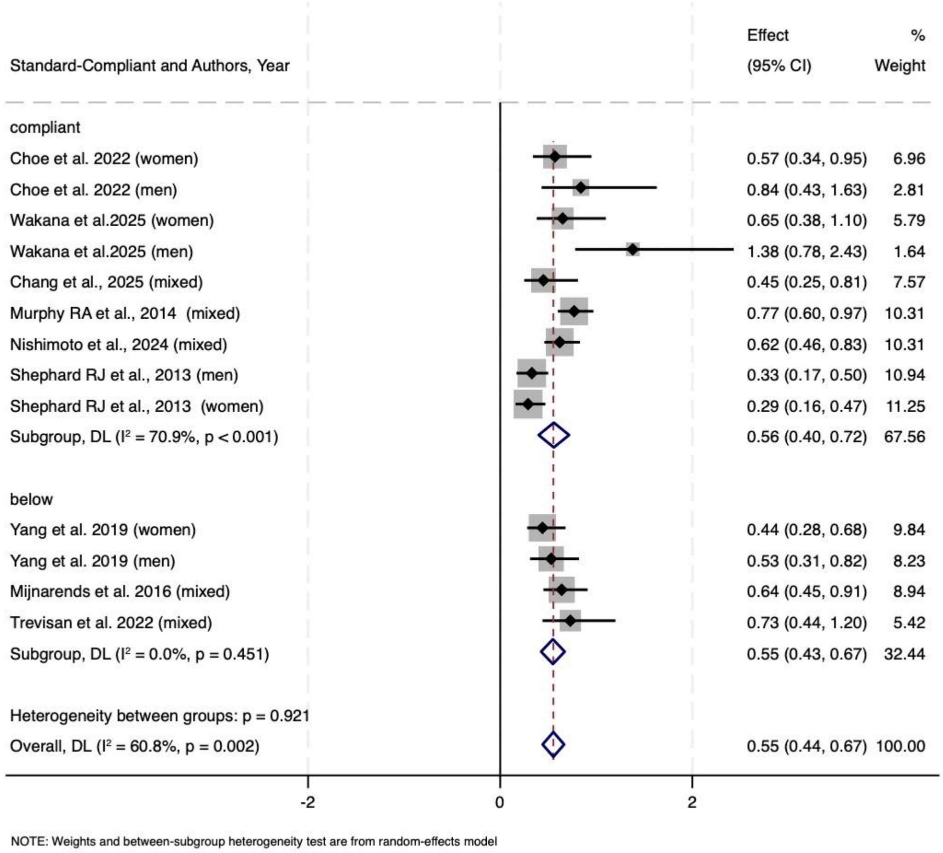


**Figure S6: Forest plot of subgroup analysis by compliance with international physical activity guidelines (WHO/ACSM)**

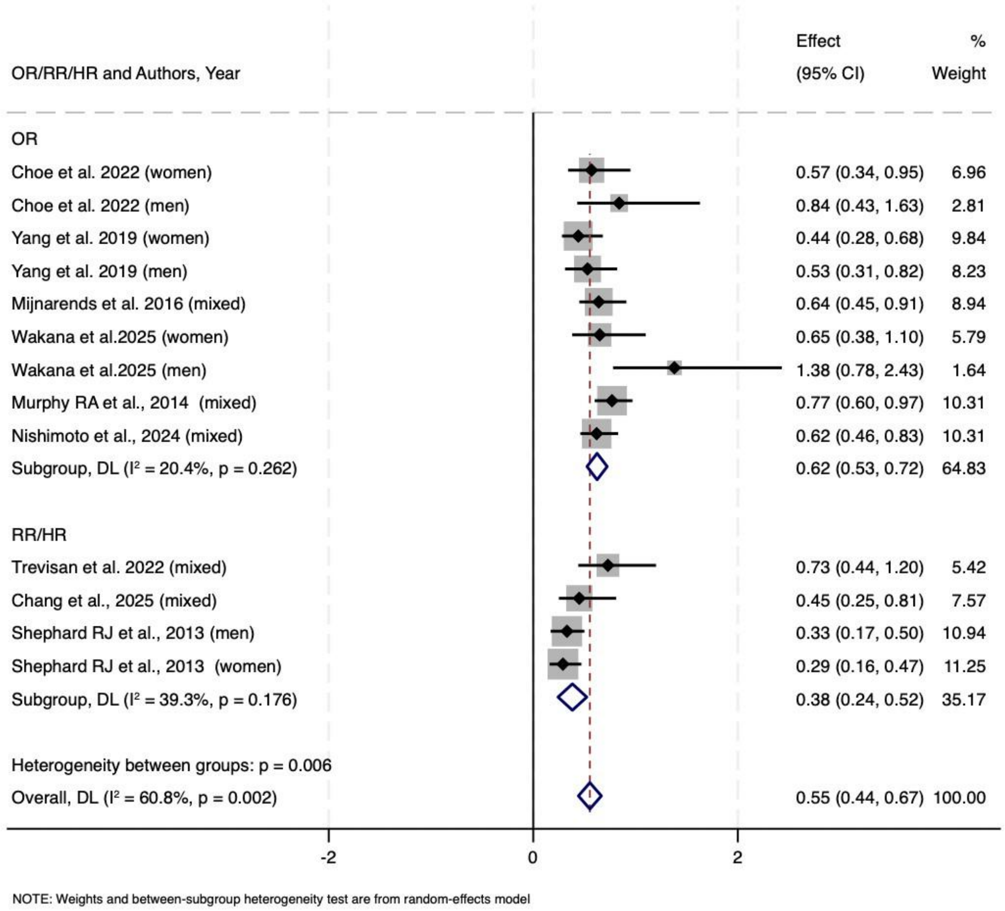


**Figure S7: Forest plot of the subgroup analysis stratified by effect measure type (OR vs. RR/HR)**

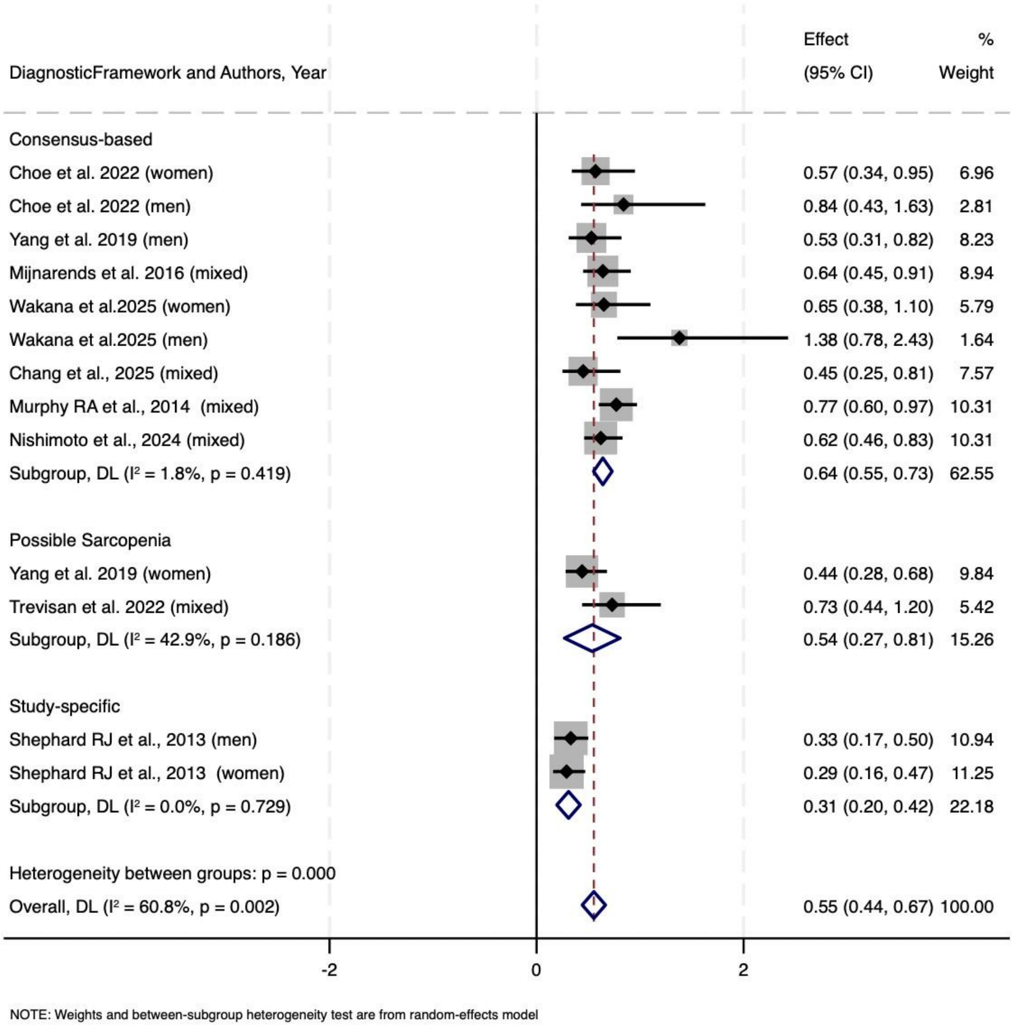


**Figure S8: Forest plot of the subgroup analysis stratified by diagnostic framework**

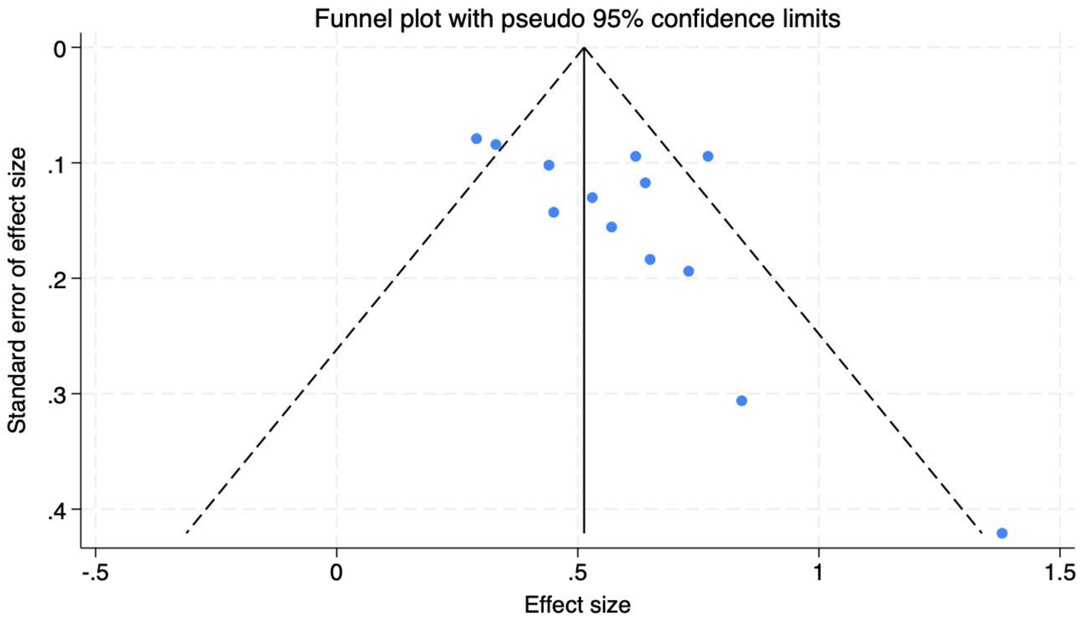


**Figure S9: Standardized Funnel plot corresponding to the main random-effects meta-analysis**

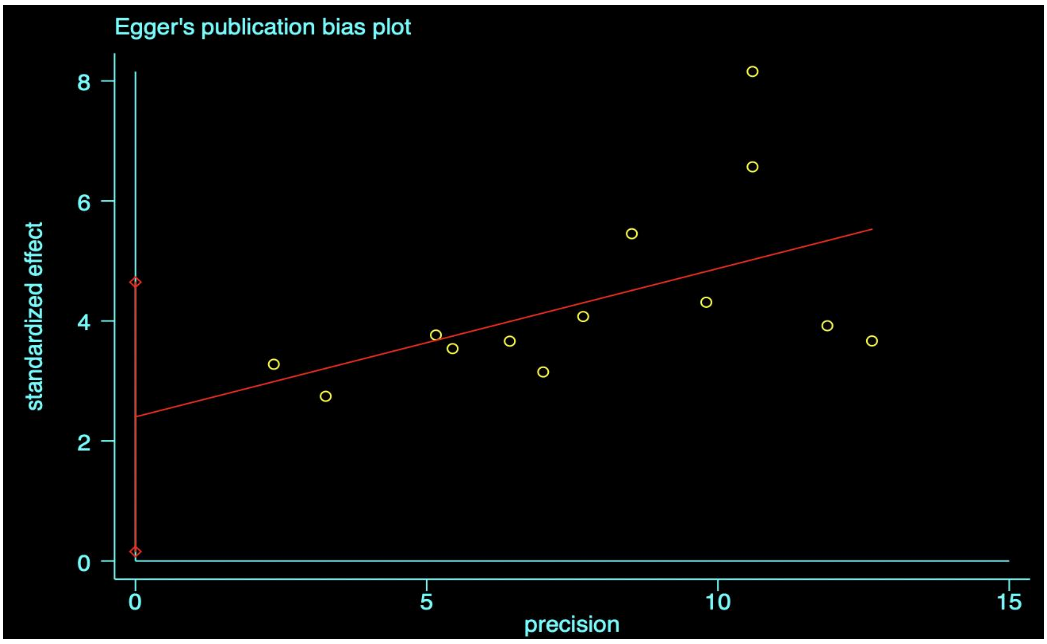


**Figure S10: Standardized Egger's regression test corresponding to the main random-effects meta-analysis**

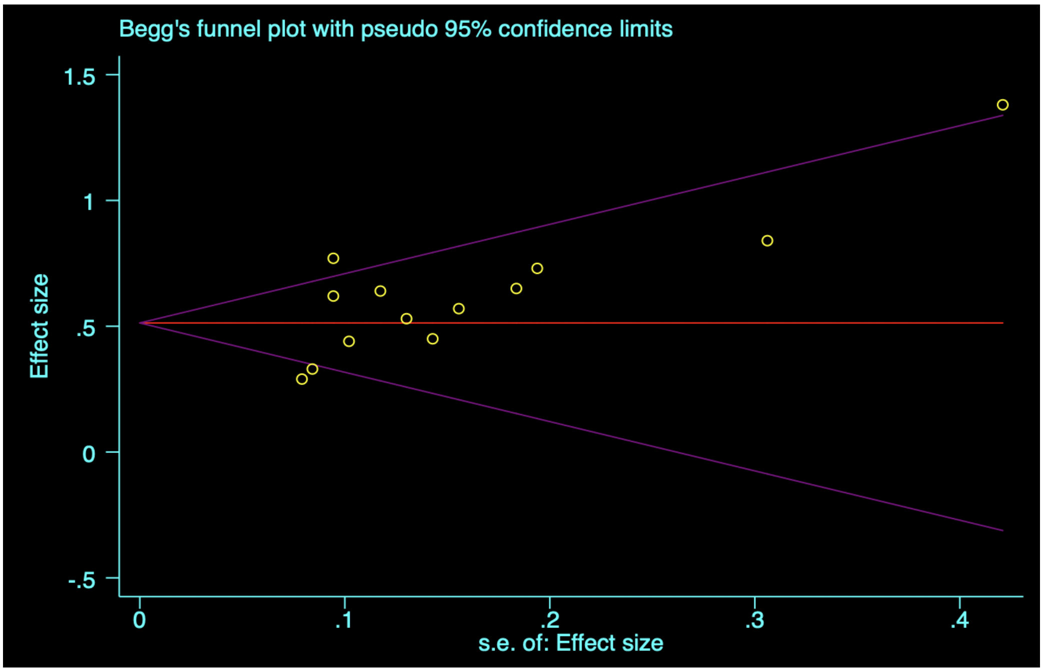


**Figure S11: Standardized Begg's rank correlation test corresponding to the main random-effects meta-analysis**

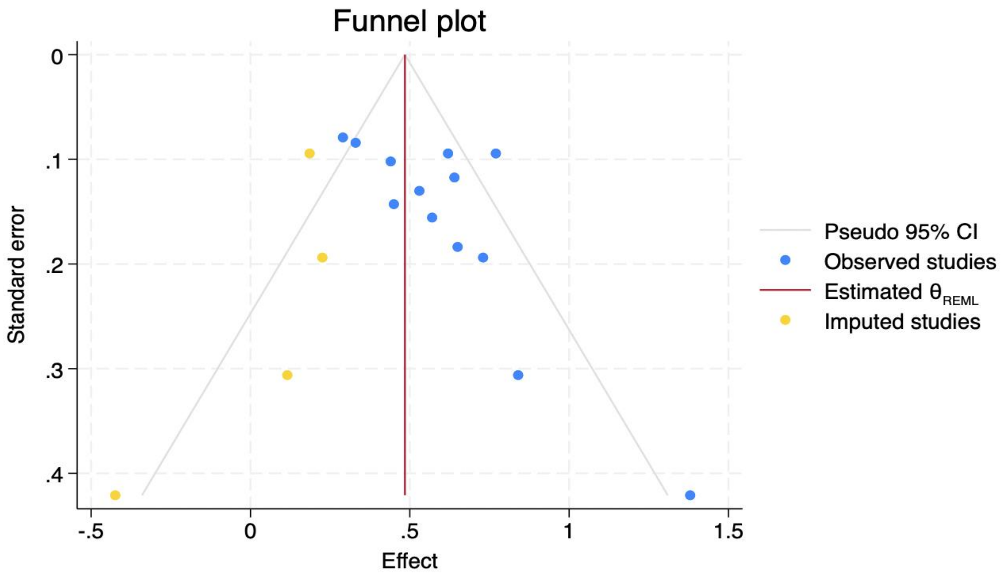


**Figure S12: Trim-and-fill Funnel plot corresponding to the main random-effects meta-analysis**

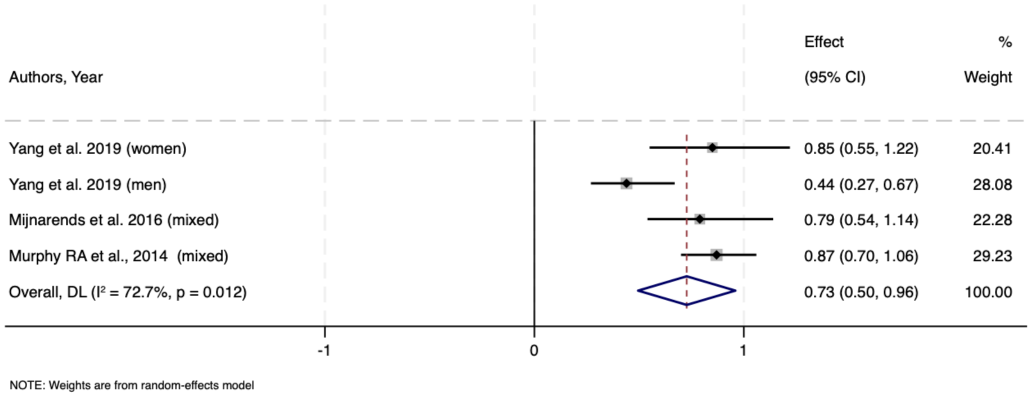


**Figure S13: Forest plot of a random effects meta-analysis including 4 risk estimates of sarcopenia for a moderate versus low level of HPA**

# PRISMA 2020 Checklist

| Section and Topic | Item # | Checklist item | Location where item is reported |
|---|---|---|---|
| **TITLE** | | | |
| Title | 1 | Identify the report as a systematic review. | Title |
| **ABSTRACT** | | | |
| Abstract | 2 | See the PRISMA 2020 for Abstracts checklist. | Abstract |
| **INTRODUCTION** | | | |
| Rationale | 3 | Describe the rationale for the review in the context of existing knowledge. | Introduction |
| Objectives | 4 | Provide an explicit statement of the objective(s) or question(s) the review addresses. | Introduction |
| **METHODS** | | | |
| Eligibility criteria | 5 | Specify the inclusion and exclusion criteria for the review and how studies were grouped for the syntheses. | Methods - Inclusion and exclusion criteria |
| Information sources | 6 | Specify all databases, registers, websites, organisations, reference lists and other sources searched or consulted to identify studies. Specify the date when each source was last searched or consulted. | Methods - Literature search |
| Search strategy | 7 | Present the full search strategies for all databases, registers and websites, including any filters and limits used. | Methods - Literature search; Appendix 1 |
| Selection process | 8 | Specify the methods used to decide whether a study met the inclusion criteria of the review, including how many reviewers screened each record and each report retrieved, whether they worked independently, and if applicable, details of automation tools used in the process. | Methods - Inclusion and exclusion criteria |
| Data collection process | 9 | Specify the methods used to collect data from reports, including how many reviewers collected data from each report, whether they worked independently, any processes for obtaining or confirming data from study investigators, and if applicable, details of automation tools used in the process. | Methods - Data extraction |

# PRISMA 2020 Checklist

| Section and Topic | Item # | Checklist item | Location where item is reported |
|---|---|---|---|
| Data items | 10a | List and define all outcomes for which data were sought. Specify whether all results that were compatible with each outcome domain in each study were sought (e.g. for all measures, time points, analyses), and if not, the methods used to decide which results to collect. | Methods - Inclusion and exclusion criteria |
| | 10b | List and define all other variables for which data were sought (e.g. participant and intervention characteristics, funding sources). Describe any assumptions made about any missing or unclear information. | Methods - Data extraction |
| Study risk of bias assessment | 11 | Specify the methods used to assess risk of bias in the included studies, including details of the tool(s) used, how many reviewers assessed each study and whether they worked independently, and if applicable, details of automation tools used in the process. | Methods - Study quality |
| Effect measures | 12 | Specify for each outcome the effect measure(s) (e.g. risk ratio, mean difference) used in the synthesis or presentation of results. | Methods - Data analysis |
| Synthesis methods | 13a | Describe the processes used to decide which studies were eligible for each synthesis (e.g. tabulating the study intervention characteristics and comparing against the planned groups for each synthesis (item #5)). | Methods - Data analysis |
| | 13b | Describe any methods required to prepare the data for presentation or synthesis, such as handling of missing summary statistics, or data conversions. | Methods - Data extraction ; Data analysis |
| | 13c | Describe any methods used to tabulate or visually display results of individual studies and syntheses. | Methods - Data analysis |
| | 13d | Describe any methods used to synthesize results and provide a rationale for the choice(s). If meta-analysis was performed, describe the model(s), method(s) to identify the presence and extent of statistical heterogeneity, and software package(s) used. | Methods - Data analysis |
| | 13e | Describe any methods used to explore possible causes of heterogeneity among study results (e.g. subgroup analysis, meta-regression). | Methods - Data analysis |

# PRISMA 2020 Checklist

| Section and Topic | Item # | Checklist item | Location where item is reported |
|---|---|---|---|
| | 13f | Describe any sensitivity analyses conducted to assess robustness of the synthesized results. | Methods - Data analysis |
| Reporting bias assessment | 14 | Describe any methods used to assess risk of bias due to missing results in a synthesis (arising from reporting biases). | Methods - Data analysis |
| Certainty assessment | 15 | Describe any methods used to assess certainty (or confidence) in the body of evidence for an outcome. | Methods - Study quality |
| **RESULTS** | | | |
| Study selection | 16a | Describe the results of the search and selection process, from the number of records identified in the search to the number of studies included in the review, ideally using a flow diagram. | Results - Study selection process |
| | 16b | Cite studies that might appear to meet the inclusion criteria, but which were excluded, and explain why they were excluded. | Results - Study selection process |
| Study characteristics | 17 | Cite each included study and present its characteristics. | Results - Characteristics of included studies ; Table S1 |
| Risk of bias in studies | 18 | Present assessments of risk of bias for each included study. | Results - Research quality evaluation ; Table S2 |
| Results of individual studies | 19 | For all outcomes, present, for each study: (a) summary statistics for each group (where appropriate) and (b) an effect estimate and its precision (e.g. confidence/credible interval), ideally using structured tables or plots. | Results - Primary outcome ; |

| Section and Topic | Item # | Checklist item | Location where item is reported |
|---|---|---|---|
| | | | Table S1 |
| Results of syntheses | 20a | For each synthesis, briefly summarise the characteristics and risk of bias among contributing studies. | Results - Primary outcome |
| | 20b | Present results of all statistical syntheses conducted. If meta-analysis was done, present for each the summary estimate and its precision (e.g. confidence/credible interval) and measures of statistical heterogeneity. If comparing groups, describe the direction of the effect. | Results - Primary outcome |
| | 20c | Present results of all investigations of possible causes of heterogeneity among study results. | Results - Primary outcome |
| | 20d | Present results of all sensitivity analyses conducted to assess the robustness of the synthesized results. | Results - Primary outcome |
| Reporting biases | 21 | Present assessments of risk of bias due to missing results (arising from reporting biases) for each synthesis assessed. | Results - Primary outcome |
| Certainty of evidence | 22 | Present assessments of certainty (or confidence) in the body of evidence for each outcome assessed. | Results - Research quality evaluation |
| **DISCUSSION** | | | |
| Discussion | 23a | Provide a general interpretation of the results in the context of other evidence. | Discussion |
| | 23b | Discuss any limitations of the evidence included in the review. | Discussion |
| | 23c | Discuss any limitations of the review processes used. | Discussion |
| | 23d | Discuss implications of the results for practice, policy, and future research. | Discussion; Conclusions |

# PRISMA 2020 Checklist

| Section and Topic | Item # | Checklist item | Location where item is reported |
|---|---|---|---|
| **OTHER INFORMATION** | | | |
| Registration and protocol | 24a | Provide registration information for the review, including register name and registration number, or state that the review was not registered. | Abstract; Methods |
| | 24b | Indicate where the review protocol can be accessed, or state that a protocol was not prepared. | Abstract; Methods |
| | 24c | Describe and explain any amendments to information provided at registration or in the protocol. | Methods |
| Support | 25 | Describe sources of financial or non-financial support for the review, and the role of the funders or sponsors in the review. | Funding |
| Competing interests | 26 | Declare any competing interests of review authors. | Disclosure of interest |
| Availability of data, code and other materials | 27 | Report which of the following are publicly available and where they can be found: template data collection forms; data extracted from included studies; data used for all analyses; analytic code; any other materials used in the review. | Data availability |

*From:* Page MJ, McKenzie JE, Bossuyt PM, Boutron I, Hoffmann TC, Mulrow CD, et al. The PRISMA 2020 statement: an updated guideline for reporting systematic reviews. BMJ 2021;372:n71. doi: 10.1136/bmj.n71
